# Gallstone ileus: Unusual complication of cholelithiasis: A case report

**DOI:** 10.1016/j.amsu.2022.103476

**Published:** 2022-03-04

**Authors:** Abdihamid Mohamed Ali, Sadettin ER

**Affiliations:** a Department of General Surgery, Mogadishu Somali Turkey, Recep Tayyip Erdogan Training and Research Hospital, Somalia; b Department of General Surgery, Ankara City Hospital, Ankara, Turkey

**Keywords:** Gallstone ileus, Intestinal obstruction, Enterolithotomy, CT, Computed Tomography, GB, Gallbladder, GI, Gastro-intestinal

## Abstract

**Introduction and importance:**

Gallstone ileus is a rare cause of intestinal obstruction with a high morbidity and mortality rate, which is often linked to delayed or misdiagnosed intestinal obstruction. Gallstone ileus requires a high index of suspicion to diagnose.

**Case presentation:**

This report describes a 55-year-old male who presented the case of gallstone ileus with four years history of gallstone disease, emergency explorative laparotomy was done, enterotomy and stone extraction from the small bowel, the post-operative patient was uneventful and was discharged after full enteral tolerance.

**Clinical discussion:**

CT scanning has become increasingly important as a diagnostic tool, with a sensitivity of 93% and its use has increased in recent years, In the case of patients with gallstone ileus, simple enterolithotomy is both safe and effective.

**Conclusions:**

Gallstone Ileus is an uncommon complication of gallstone disease, most commonly seen in females in advanced age, our case report presents young adult male and high index suspicion in diagnosis and urgent intervention is mandatory for better outcome of the patients.

## Highlights

• Generally this condition is seen in female with advanced age, but this case, reports atypical presentation of the disease according patient age and gender.
• Will raise awareness among patients who have a delayed presentation of the disease and acquire challenges associated to gallstones, such as gallstone illeus.
• To illustrate all necessary requirement of diagnosis and management of disease. Including classic radiological features and relevant intra-operative images.
• Summarize the surgical techniques performed to management gallstone ileus.

## 1 Background

Gallstone ileus is an uncommon condition caused by gallstone impaction in the gastrointestinal tract lumen that affects 0.3% to 0.5% of cholelithiasis patients. Gallstone ileus occurs in 1%–4% of all cases of mechanical intestinal obstruction and is rarely detected before surgery [1].

Because symptoms may be intermittent and studies may fail to establish the reason that causes the obstruction, the diagnosis is sometimes delayed.

A strong index of suspicion is required for diagnosis, As a result of this circumstance, gallstone ileus still has a high rate of morbidity and mortality [2].

Only approximately half of these individuals have a prior history of gallbladder illness, which is a significant clinical antecedent. It's crucial to remember that small intestine is the cause of small intestine. Only 4% of patients under the age of 65 have bowel obstruction.

Patients under 65 years of age, while it climbs to 25% in those beyond 65 years of age [3].

The gallstone's inflammation and pressure create erosion of the gallbladder wall, resulting in the creation of a fistula between the gallbladder and the nearby and adherent segment of the gastrointestinal tract, allowing the gallstone to pass through.

However, due to the poor general status of ileus patients, physicians must choose between immediate one-stage or two-stage closure of the cholecystointestinal fistula or waiting for natural closure [4].

## 2 Case presentation

A 55 -year-old man was admitted to the emergency department with a 7 days history of abdominal pain, intermittent nausea, vomiting, and constipation for the last 2 days. Physical examination revealed a soft, non-tender abdomen, but bowel sounds were present on abdominal auscultation.

**Fig. 1:**
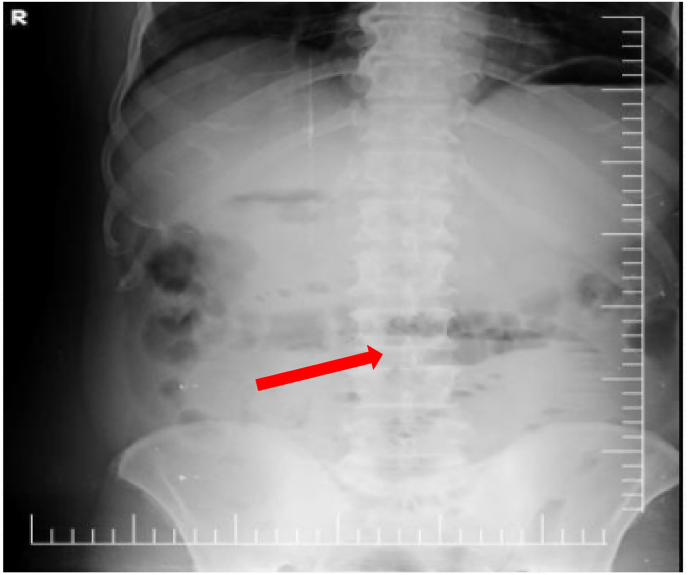
Erect abdominal plain film shows mild dilated small bowel loops with air-fluid levels in bowel and stomach.

**Fig. 2:**
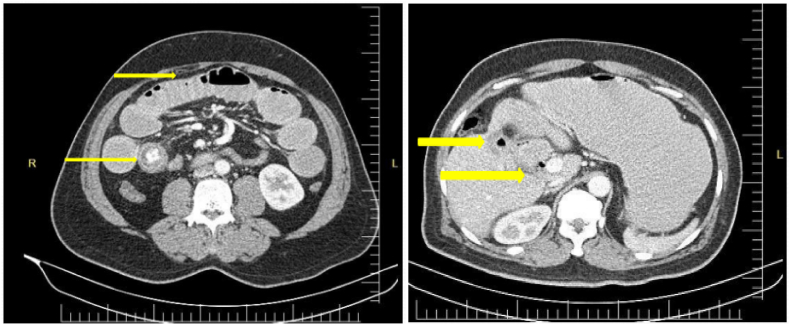
Computed Tomography scan revealed dilated bowel loops and concentric circles in the small intestine consistent with a gallstone ileus and decompressed GB and air in GB, verifying that a stone was released (yellow arrow). (For interpretation of the references to colour in this figure legend, the reader is referred to the Web version of this article.)

The erect abdominal plain film showed mildly dilated loops of the small bowel with air-fluid levels and distention of the stomach (Fig. 1). Abdominal CT has performed the Rigler's triad of findings of gallstone ileus was Present. Dilated loops of the small bowel, the air in the gallbladder and the biliary tree (Fig. 2), and an ectopic stone in the jejunum (Fig. 2) were demonstrated.

**Fig. 3:**
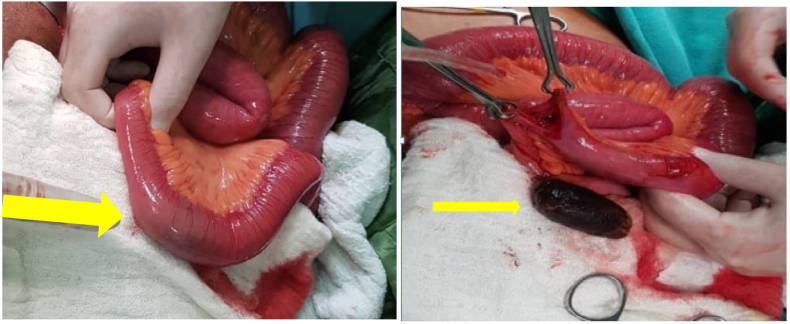
Demonstrates intra luminal gallstone inside small bowel and was removed and impacted stone (arrow) with babcock holding at the enterotomy edges of small intestine.

The patient was planned urgent explorative laparotomy, Intraoperatively upon exploration proximal bowel was markedly distended, an impacted gallstone was seen in the jejunum, and Stone was milked to more distal where an enterotomy was made to reveal a large gallstone (Fig. 3).

The gallstone was removed and the enterotomy repair was done. The patient was discharged from the hospital in post-operative several days with full recovery and follow in polyclinic was uneventful.

## 3 Discussion

The morbidity and mortality rate of gallstone ileus remain very high, partially because of misdiagnosis and delayed diagnosis. Thus, early diagnosis and treatment may help to lower mortality rates.

Plain abdomen radiographs can help in diagnosis, although its sensitivity is low, ranging from 40 to 70% [5]. With a sensitivity of 93%, CT scanning has become more widely available as a diagnostic method [6].

The optimal management of acute GI is controversial and can be divided into three subgroups 1) enterolithotomy alone, 2) one-stage procedure of enterolithotomy, cholecystectomy and fistula closure and 3) two-stage procedure of enterolithotomy with an interval cholecystectomy and fistula closure. However as most of patient presents in acute settings additional time and added risk may not be warranted [7].

In the case of patients with gallstone ileus, simple enterolithotomy is both safe and effective [8].

When dealing with a case of small bowel obstruction, keep gallstone ileus in mind, especially if the patient is old and the diagnosis is easily overlooked. The mainstay of treatment is early surgical surgery. Based on these findings, two-stage surgery is now the conventional treatment for gallstone ileus and small intestine impaction [9].

## 4 Conclusion

The advanced manifestation of gallstone disease decreased after the universal use of laparoscopic cholecystectomy for the treatment of gallstone disease.

Females in their later years are more likely to manifest with gallstone ileus. Our case study focused on a young adult male who had gallstone ileus, demonstrating the need of considering this complication in a young adult with a lengthy history of untreated gallstone illness. The surgical treatment choice for gallstone ileus is still debatable. Because of its minimal risk of complications, enterotomy with stone extraction alone is still the most popular surgical procedure.

This work has been reported in line with the SCARE 2020 criteria [10].

## Availability of data and materials

The author declare that all data in this article are available within the article.

## Ethics approval and consent to participate

Not applicable.

## Consent for publication

For the publication of this case report and any accompanying pictures, the patient provided written informed consent. A copy of the written consent is available for review by the Editor-in-Chief of this journal on request.

## Availability of data and materials

The author declare that all data in this article are available within the article.

## Funding

Not applicable.

## Provenance and peer review

Not commissioned, externally peer-reviewed.

## Guarantor

Dr Abdihamid Mohamed Ali.

## CRediT authorship contribution statement

Dr Abdihamid Mohamed and Ass. Prof Sadettin ER have made substantial contributions to the concept and design of the case report. All authors read and approved the final manuscript.

## Research registration number

Not applicable.

## Declaration of competing interest

The authors declare that they have no competing interests.
